# S100B Levels Following Administration of Thiopental and Fentanyl Midazolam Combination in Patients Undergoing Mechanical Ventilation: A Comparative Study

**DOI:** 10.5812/ijpr-157513

**Published:** 2025-05-20

**Authors:** Sara Salarian, Mohammad Sistanizad, Mohammad Gharehbeglou, Keyhan Poorali, Bahador Bagheri

**Affiliations:** 1 Department of Critical Care Medicine, Imam Hossein Medical and Educational Center, Shahid Beheshti University of Medical Sciences, Tehran, Iran; 2 Prevention of Cardiovascular Disease Research Center, Shahid Beheshti University of Medical Sciences, Tehran, Iran; 3 Department of Anesthesiology and Critical Care Medicine, School of Medicine, Shahid Beheshti University of Medical Sciences, Tehran, Iran; 4 Department of Anesthesiology and Critical Care Medicine, School of Medicine, Zahedan University of Medical Sciences, Zahedan, Iran; 5 Department of Surgery, Kowsar Hospital, Semnan University of Medical Sciences, Semnan, Iran; 6 Cancer Research Center, Semnan University of Medical Sciences, Semnan, Iran; 7 Center for Molecular Cardiology, University of Zurich, Schlieren, Switzerland

**Keywords:** Thiopental, S-100B, Brain Injury, ICU, Midazolam

## Abstract

**Background:**

To compare the effects of fentanyl combined with midazolam versus thiopental on serum levels of S100B in critically ill patients with brain injury.

**Methods:**

Eighty-five patients who underwent neurosurgical interventions and required mechanical ventilation were included in the study. The Sequential Organ Failure Assessment (SOFA) score, duration of ventilation, length of intensive care unit (ICU) stay, and serum levels of S100B were measured and compared at baseline, day 3, and day 5 of the study.

**Results:**

Seventy out of the 85 selected patients were randomized. There were no significant differences in the levels of S100B at admission (P = 0.7) and on day 3. However, on day 5, S100B levels were significantly lower in the thiopental group compared to the fentanyl plus midazolam group (P = 0.03). Although the duration of ventilation was shorter in the thiopental group, the difference was not statistically significant (P = 0.06). Additionally, the length of ICU stay was shorter for patients who received thiopental (214 hours vs. 209 hours, P = 0.4).

**Conclusions:**

Our study demonstrated that both thiopental and fentanyl combined with midazolam could reduce S100B levels in patients with traumatic brain injuries. Furthermore, patients receiving thiopental experienced a shorter ICU stay and duration of ventilation.

## 1. Background

The intensive care unit (ICU) is designed for patients recovering from anesthesia and those requiring critical care. Critically ill patients may suffer from trauma, stroke, sepsis, traumatic brain injury (TBI), and brain surgery (1). Neurological complications are major concerns in these patients. Therefore, the judicious use of medications and techniques is vital to improve their clinical status. Several neuroprotective medications are used to enhance clinical outcomes and reduce the length of ICU stay (2, 3). Barbiturates, including thiopental, are common intravenous anesthetics used in ICU settings. Thiopental, as a barbiturate, is generally considered a neuroprotective agent. It can reduce the cerebral metabolic rate of oxygen (CMRO2) and cerebral blood flow (CBF), and more importantly, it may improve clinical outcomes and reduce mortality (4, 5). Fentanyl combined with midazolam is another common regimen with favorable effects in ICU patients (6). Currently, various biomarkers are used to estimate the prognosis and risk stratification of brain injuries (7). Upon brain injury, the integrity of the blood-brain barrier (BBB) is compromised, allowing a rise in brain biomarker levels (7, 8). In recent years, calcium-binding protein S100B and neuron-specific enolase (NSE) have gained prominence as common biomarkers in brain injuries. S100B is mainly expressed in astrocytes and the peripheral nervous system (9). Following brain injury, S100B is released into the cerebrospinal fluid (CSF) and bloodstream through the arachnoid villi. Elevated levels of S100B are usually associated with mortality and poor prognosis (10, 11). In addition to ICU settings, some studies have found a role for S100B in electroconvulsive therapy, indicating that increased S100B levels are associated with neuronal distress (12). Current evidence regarding the effects of thiopental on S100B levels is inconclusive and remains debatable. Interestingly, some experimental data indicate that thiopental may cause a marked increase in S100B levels in the CSF, while having no effect on S100B levels in the blood (13).

## 2. Objectives

Excessive use of sedatives and analgesics may lead to respiratory depression, hemodynamic instability, and gastrointestinal discomfort. Therefore, new drugs or drug combinations should be studied to achieve more beneficial effects. This trial was designed to compare the effects of thiopental with fentanyl combined with midazolam on S100B levels in patients admitted to the ICU.

## 3. Methods

### 3.1. Study Design

The study was conducted at Imam Hossein Hospital, Tehran, Iran. Informed consent was obtained from all participants or their legal representatives. Inclusion criteria included neurosurgical interventions in patients requiring mechanical ventilation, aged 18 to 70 years. Patients with a history of liver disorders [liver enzyme abnormalities > 3 times the upper limit of normal (ULN)], severe renal failure (creatinine increase × 3 or creatinine ≥ 4 mg/dL and urine output < 0.3 mL/kg/h × 24 h), history of drug hypersensitivity, systolic blood pressure (SBP) < 90 mm Hg, heart rate (HR) < 60/min, anemia, malignancy, inflammatory conditions and systemic infections, pneumonic operations, video-assisted thoracic surgery (VATS), and patients with a positive COVID-19 test (PCR) were excluded from the study. The study was implemented according to the protocols of the study hospital and was an open-label trial. Eighty-five patients were randomly divided 1:1 into two groups: The fentanyl plus midazolam group and the thiopental group, using permuted block randomization. Serum levels of S100B were measured at admission, day 3, and day 5. Fentanyl combined with midazolam was administered at 0.06 to 0.2 mg/kg/h (Caspian Pharmaceuticals, Iran) (14, 15). Patients received thiopental (PANPHARMA, France) at 0.3 to 3 mg/kg/h as a maintenance dose. Infusion rates were adjusted to achieve and maintain a ramsay sedation score (RSS) of 3 to 4 every 10 minutes (16). Additionally, a Behavioral Pain Scale (BPS) score ≤ 4 was used to evaluate analgesia levels (17). A spontaneous breathing trial (SBT) was used for weaning (18). In our previous works, the Rapid Shallow Breathing Index (RSBI) was used for successful weaning (19). Patients were followed until ICU discharge. The Sequential Organ Failure Assessment (SOFA) score and the demographic data of patients, including age, sex, and history of previous diseases, were recorded and compared between groups.

### 3.2. Efficacy and Safety Assessment

The primary endpoint was the change in serum levels of S100B in both treatment groups. Levels of S100B were measured at baseline, day 3, and day 5 of treatment using an enzyme-linked immunosorbent assay (ELISA) (DiaMetra, UK). Briefly, standards and samples were added to each well and incubated at 37°C for 2 hours. After incubation, 100 µL of working biotin conjugate antibody was added to each well and incubated at 37°C for 1 hour. Subsequently, 100 µL of working streptavidin-HRP was added to each well and incubated at 37°C for 1 hour. Then, 100 µL of substrate solution was added to each well, and following a 15- to 20-minute incubation at 37°C, the reaction was stopped using stop solution, and absorption was measured at 450 nm using a microplate reader (Stat Fax 2100 Awareness, AZ, USA). The duration of ventilation, length of ICU stay, and untoward effects of medications such as respiratory depression, hypotension, and bradycardia were the secondary endpoints. Patients were continuously monitored for sedation, analgesia, and adverse effects of drugs. Patients were excluded from the study due to safety concerns.

### 3.3. Data Analysis

The total number of 85 patients was calculated for randomization according to the assumption of a 20% dropout rate among study patients, with an 80% power and an anticipated effect size of 0.1. A significance level of 0.05 for changes in S100B levels with a sample size of 70 patients was estimated. Student *t*-test, chi-square test, and Fisher’s exact test were used for data analysis due to the normal distribution of collected data. A P-value of < 0.05 was considered statistically significant. Analysis was performed using SPSS software version 21.0 (Chicago, USA).

## 4. Results

### 4.1. Baseline Characteristics

**Figure 1.**
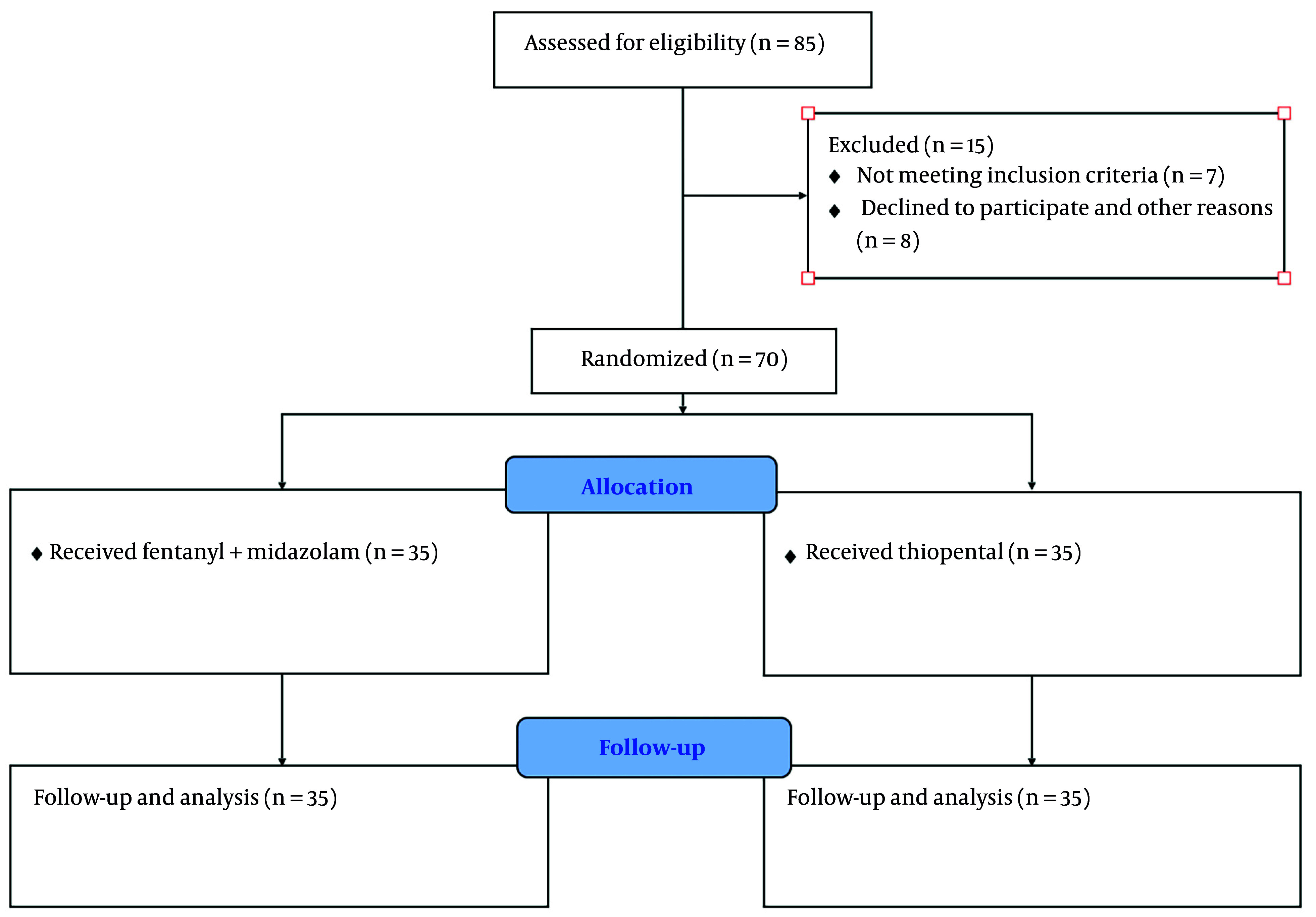
Consort diagram detailing study subjects

Of the 85 study subjects included, 15 patients did not receive the treatments (7 met the exclusion criteria, 6 declined to participate, and 2 for other reasons). Patients were studied from September 2021 to October 2022. No patient was lost to follow-up, and all 70 participants were observed over the 5 days and until ICU discharge. The mean age was 55 years, with a predominance of females (52% vs 48%). Table 1 shows the clinical characteristics of the study participants. No significant differences were observed at baseline. Patient flow is shown in Figure 1. 

**Table 1.** Baseline Characteristics of Two Study Groups ^a^

Characteristics	Fentanyl + Midazolam (n = 35)	Thiopental (n = 35)	P-Value
**Age; y**	53.41 ± 5.31	56.62 ± 9.13	0.3
**Age; y (range)**	21 - 66	20 - 68	0.4
**Female**	19 (54)	18 (51)	0.4
**Body weight; kg**	79 ± 1.82	81 ± 6.91	0.3
**Reason for admission **			
**Trauma**	11 (35)	15 (42.8)	0.2
**Surgery**	14 (40)	15 (42.8)	0.3
**Other**	10 (28.5)	5 (14.2)	0.6
**GCS**	4 ± 1	3 ± 1	0.7
**Total SOFA score**	6 ± 2	7 ± 1	0.0
**Hemoglobin; g/L**	103 ± 3.92	101 ± 3.21	0.5
**Total WBC count; 10** ^**9**^ **/L**	7.2 ± 3.33	7.3 ± 2.93	0.8
**Platelets; 10** ^**9**^ **/L**	211 ± 111	220 ± 109	0.7
**SBP; mmHg**	131 ± 6.21	119 ± 2.13	0.5
**DBP; mmHg**	61 ± 5.92	60 ± 6.43	0.4
**Respiratory rate; breaths/min**	15 ± 1.12	18 ± 2.33	1.0
**Heat rate; beats/min**	73 ± 1.01	70 ± 1.11	0.3

^a^ Values are expressed as No. (%) or mean ± SD.

### 4.2. Clinical Outcomes

The target sedation level (RSS = 3 - 4) was achieved in both study groups. No significant differences were noted at specific time intervals (P = 0.7). Additionally, the target analgesia level (BPS ≤ 4) was reached in both groups, and the differences were not significant (P = 0.8). As indicated in Table 2, baseline S100B levels were 9.08 ± 6.77 ng/L in the fentanyl plus midazolam group and 9.31 ± 3.73 ng/L in the thiopental group (P = 0.1). Both treatment modalities decreased S100B levels on day 3; however, the differences were not significant. On day 5, a significant difference was noted in S100B levels in favor of patients who received thiopental (5.03 ± 1.9 ng/L vs 6.98 ± 2.12 ng/L, P = 0.03). Notably, subgroup analysis found no difference in S100B levels among patients with diabetes at different time intervals in both study groups. Although the duration of mechanical ventilation was shorter in the thiopental group, the differences were not significant; 6.1 ± 1.3 days vs 5.1 ± 1.8 days (P = 0.06). Furthermore, as presented in Table 2, although the ICU stay (period between ICU admission and ICU discharge) was shorter in the thiopental group, the difference failed to reach a significant level. The ICU stay was 214 ± 11 hours in the fentanyl plus midazolam group and 209 ± 21 hours in the thiopental group (P = 0.4).

**Table 2.** Comparison Between Two Study Groups; S100B Levels, Duration of Ventilation, Intensive Care Unit Stay, and Side Effects of the Treatments ^a^

Variables	Fentanyl + Midazolam (n = 35)	Thiopental (n = 35)	P-Value
**S100B; µg/L**			
Baseline	9.08 ± 6.77	9.31 ± 3.73	0.7
Day 3	7.61± 2.32	7.90 ± 2.43	0.6
Day 5	6.98 ± 2.12	5.03 ± 1.92	0.03
**Duration of ventilation; d**	6.11 ± 1.38	5.11 ± 1.13	0.4
**Length of ICU stay; h**	214 ± 11	209 ± 21	0.8
**Hypotension**	8 (22.8)	6 (17.1)	0.7
**Bradycardia**	3 (8.5)	3 (8.5)	0.7

Abbreviation: ICU, intensive care unit.

^a^ Values are expressed as No. (%) or mean ± SD.

### 4.3. Adverse Effects of Treatments

We found no significant differences in the rate of bradycardia, hypotension, and respiratory depression between study groups. No patient was lost to follow-up, and no one was withdrawn due to severe adverse effects of the treatments. No deaths were reported.

## 5. Discussion

The present study demonstrated that both treatment modalities, thiopental and fentanyl combined with midazolam, were effective in decreasing S100B levels in patients with traumatic brain injuries who required mechanical ventilation. However, on day 5, patients who received thiopental showed a significant reduction in S100B levels. The mechanism by which thiopental causes a greater reduction in S100B levels compared to fentanyl plus midazolam is not clearly understood. A deeper effect on the duration of activity of GABA_A_ channels and an improvement in the CMRO2 may partially mitigate the inflammatory damage to neurons. Although a reduction was noted on day 3, the response continued to decrease on day 5. Measuring other inflammatory markers, such as cytokines or oxidative players, could provide a better understanding of the mechanisms behind these effects. The duration of mechanical ventilation and the length of ICU stay were shorter in the thiopental group. During brain injury, the integrity of the BBB is disrupted, and the physiological mechanisms responsible for restoring regular local homeostasis fail to function properly. At this stage, different compounds may be released from disrupted cells in response to damage (20, 21). However, to validate the clinical significance of such compounds, their interactions with medications should be understood and well interpreted. The neuroprotective effects of thiopental have been extensively investigated in animal studies, but they are not clearly addressed in humans. Several studies have shown that barbiturates like thiopental have a protective effect on ischemic brain injuries in various animal models (22-24). A study showed that thiopental administered before or after ischemia reduced nerve damage in gerbils, although post-ischemic thiopental treatment required larger doses (25). A study by Velle et al. indicated that barbiturate coma therapy (BCT) with thiopental could improve intracranial compensatory reserve and intracranial pressure in children with TBI (26). Although the mechanism of action of thiopental is largely due to its effects on GABA_A_ and NMDA receptors, thiopental can cause a dose-related depression of cerebral metabolic oxygen consumption rate (CMRO2) and reduce the consumption of adenosine triphosphate, which can be considered for its neuroprotective effects (4, 5). Notably, S100B is not brain-specific but is almost always present in studies about TBI and stroke. S100B can also be elevated in some carcinomas, skin diseases, and thoracic surgeries. In general, patients with TBI who have high levels of S100B are linked with poor prognosis (27-29). In line with our results, Gong et al. showed that blood levels of S100B and NSE significantly decreased in patients with hypertensive intracerebral hemorrhage (HICH) who received dexmedetomidine compared to midazolam (30).

From a pharmacological perspective, add-on therapy with medications that can decrease the total daily consumption of sedatives and opiates is considered beneficial. Previously, we demonstrated that the addition of clonidine and gabapentin to common ICU medications, such as thiopental, fentanyl, and propofol, is potentially useful in reducing the adverse effects of sedative and analgesic drugs in the ICU (31, 32). A recent study by Salarian et al. indicated that thiopental administration was associated with a greater decrease in NSE, a biomarker of traumatic brain injuries, compared to the combination of fentanyl and midazolam (33). Moreover, a new systematic review has provided evidence for the beneficial effects of hypertonic saline in patients with TBI admitted to the ICU with increased intracranial pressure (34). Interestingly, a study by Mahmoodpoor showed that a 7-day therapy with L-carnitine was not effective in reducing NSE levels or the rate of mortality in patients with TBI (35). Finally, clinicians should always be aware of hypo- or hyperkalemia in patients with TBI receiving barbiturates as part of BCT (34).

### 5.1. Conclusions

Our study demonstrated that both thiopental and fentanyl combined with midazolam effectively reduced S100B levels in patients with traumatic brain injuries. Additionally, patients receiving thiopental experienced a shorter ICU stay and duration of mechanical ventilation.

### 5.2. Limitations of the Study

One limitation of this study was the short follow-up period. It would be more beneficial to follow up with patients over a longer duration to determine any correlation between S100B levels and clinical status. Additionally, it would have been advantageous to explore the role of antioxidative markers, such as superoxide dismutase (SOD) or malondialdehyde (MDA), in addition to inflammatory cytokines.

## Data Availability

The dataset presented in the study is available on request from the corresponding author during submission or after publication.

## Clinical Trial Registration Code:

IRCT20120430009593N17.
